# Supplementary material for: CXCL4-RNA Complexes Circulate in Systemic Sclerosis and Amplify Inflammatory/Pro-Fibrotic Responses by Myeloid Dendritic Cells
Source: Int J Mol Sci. 2022 Dec 30;24(1):653. doi: 10.3390/ijms24010653 (PMC9820649; doi:10.3390/ijms24010653)
Supplement: Supplementary file 1 [file ijms-24-00653-s001.zip › ijms-2092668-supplementary.pdf]

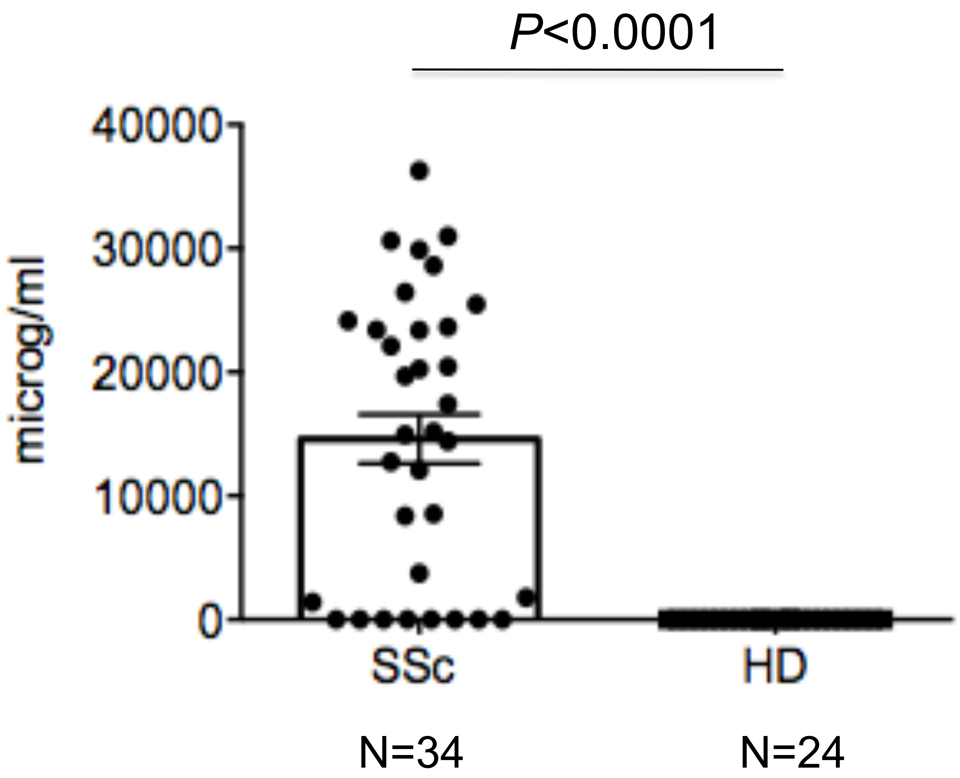

Figure S1. SSc plasma show CXCL4 in circulation. Plasma from SSc patients and healthy donors were tested by commercial ELISA for expression and concentration of CXCL4. Horizontal bars represent the means of CXCL4 concentrations, vertical bars are standard error of the mean (SEM), P value are from Mann-Whytney Test.

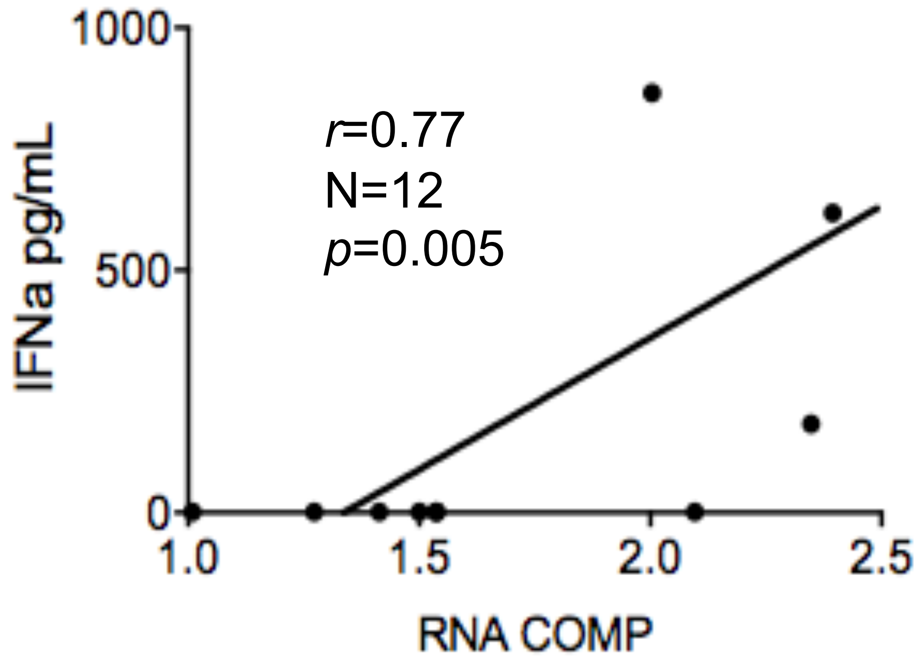

Figure S2. Presence of CXCL4-RNA complexes correlates with IFN-α in SSc plasma. Plasma from SSc patients were tested by commercial ELISA for expression and concentration of CXCL4. OD values indicating CXCL4-RNA complexes expression were plotted against IFN-α concentrations measured in the same SSc plasma. Correlation was calculated by Spearman's correlation test. Coefficient of correlation r, significance P and sample size. In this correlation we considered only the patients that were positive for expression of CXCL4-RNA complexes in their plasma.

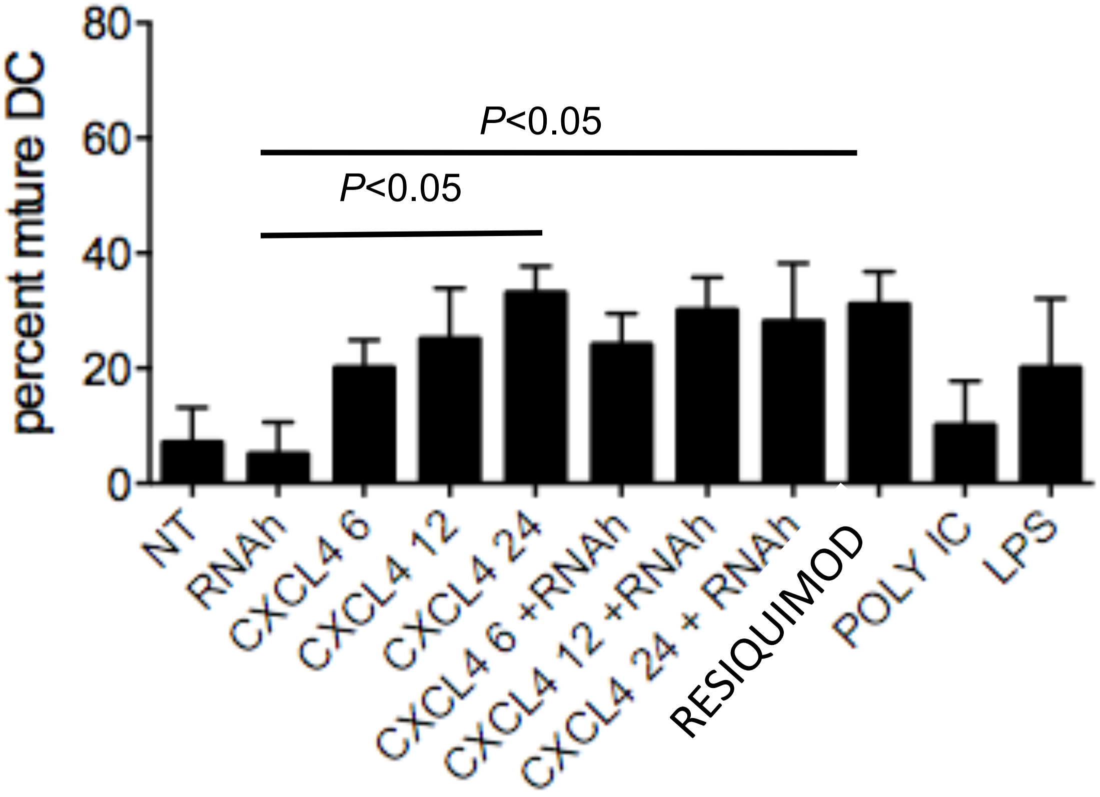

Figure S3. MDDC are stimulated to mature by CXCL4 alone in the absence of IFN-I priming. MDDC were either untreated (nt), or treated with RNA alone or CXCL4 alone or with different amounts of CXCL4-RNA complexes or with TLR agonists.MDDC maturation was evaluated by flow cytometry and is expressed as percent of double positive ($CD80^{+}CD86^{+}$) cells. Results from 5 experiments performed with MDDC derived from different donors. *P* values were by Mann-Whitney's test.

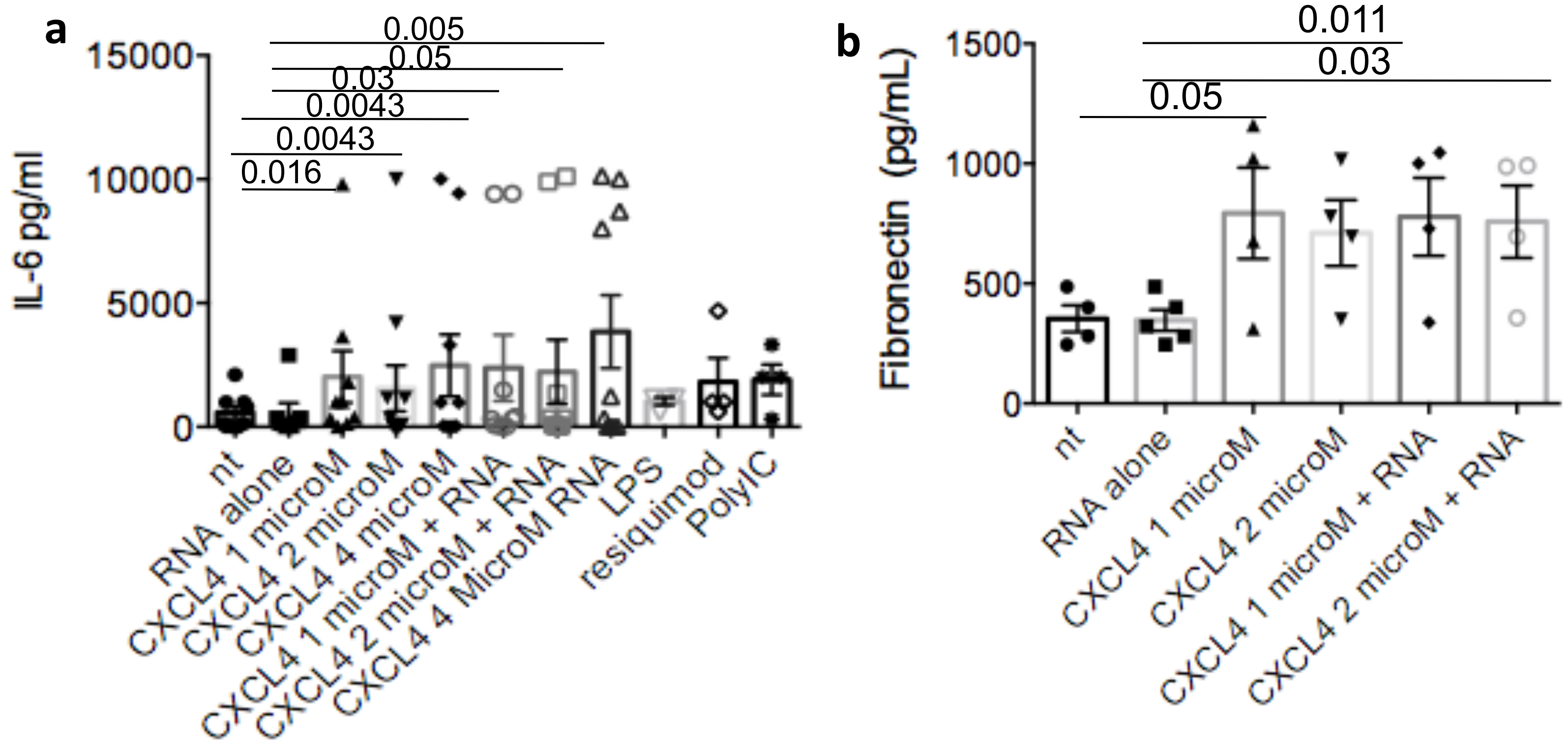

Figure S4. CXCL4 stimulates MDDC in the absence of IFN-I priming. MDDC were either untreated (nt), or treated with RNA alone or CXCL4 alone or with different amounts of CXCL4-RNA complexes or with TLR agonists. IL-6 (a) and fibronectin (b) secretion in culture supernatants were measured by ELISA after 48 hours. Results are from 5 to 8 experiments performed with MDDC derived from different donors. *P* values were by Mann-Whitney's test.

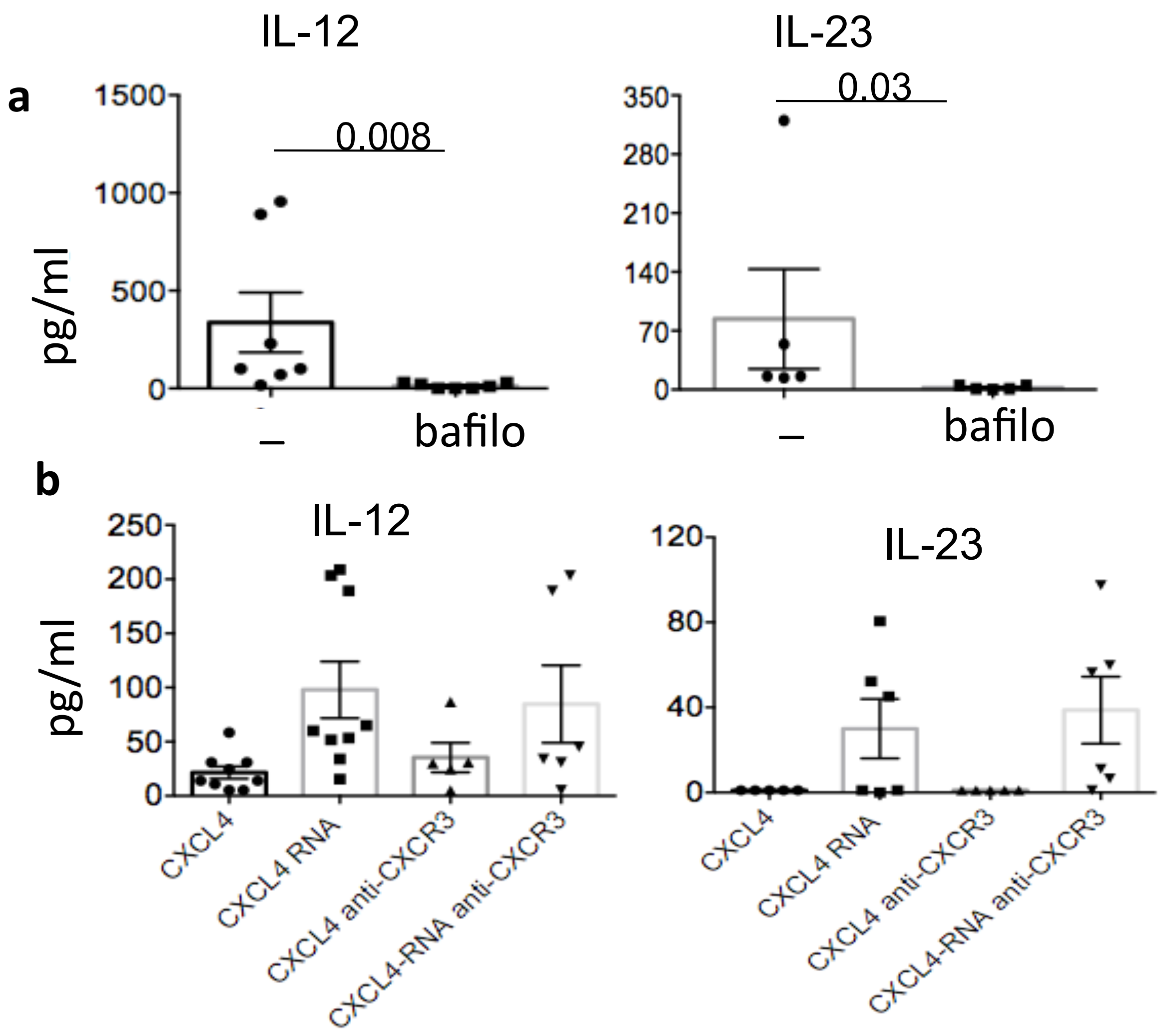

Figure S5 MDDC stimulation by CXCL4-RNA complexes is dependent on endosomal sensing TLRs and independent from CXCR3. (a) MDDC were treated with CXCL4-RNA complexes in the presence of bafilomycin to block stimulation of endosomal TLRs. Production of cytokines was evaluated by ELISA as above. **(b)** MDDC were stimulated by CXCL4-RNA complexes in the present of an anti-CXCR3 blocking antibody and cytokines measured as in **(a)**. Results are from 5 to 9 experiments (depending on the stimulus), performed with MDDC of different donors. *P* values by Mann-Whitney's test.
